# 

**Published:** 1884-01

**Authors:** 


### CONTENTS:

Art. I.—Personal Identity Established by the Teeth; the Dentist a Scientific Expert. By Richard Grady, D. D. S. of Baltimore.................... 385

Art. II.—Treatment of Fracture of the Jaw, with Critical Remarks, as sent to Prof. D. Hayes Agnew, M. D. By Thos. Bryan Gunning D. D. S..... 406

Art. III.—In Difference of Dr. S. C. Barnm ................................ 420

Art. IV.— Dies, Etc. By Haskell, Chicago.................................... 423

### EDITORIAL, ETC.

Dental Legislation................................................................ 425

